# Strain Rate Sensitivity of Tensile Properties in Ti-6.6Al-3.3Mo-1.8Zr-0.29Si Alloy: Experiments and Constitutive Modeling

**DOI:** 10.3390/ma11091591

**Published:** 2018-09-02

**Authors:** Jun Zhang, Yang Wang, Bin Zhang, Hanjun Huang, Junhong Chen, Peng Wang

**Affiliations:** 1 Institute of Systems Engineering, China Academy of Engineering Physics, Mianyang 621999, China; huanghj@caep.cn (H.H.); lxchenjh@caep.cn (J.C.); hjwangp@caep.cn (P.W.); 2 Department of Modern Mechanics, CAS Key Laboratory of Mechanical Behavior and Design of Materials, University of Science and Technology of China, Hefei 230027, China; zhb1005@mail.ustc.edu.cn

**Keywords:** tensile impact, constitutive model, adiabatic temperature rise, strain rate history

## Abstract

The complex deformation usually involves wide strain-rate change. However, few efforts have been devoted to investigate the effect of strain rate history on the tensile behavior of α + β titanium alloy. In present paper, tensile tests of Ti-6.6Al-3.3Mo-1.8Zr-0.29Si alloy were carried out under both constant and variable strain-rate conditions within the region from 10^−3^~500 s^−1^. A single stress pulse experimental technique was utilized to conduct the recovery tests. The strain-rate history effect was examined. It is found that the flow stress is independent on the strain rate history, though the alloy exhibits obvious positive strain rate sensitivity. The Taylor-Quinney coefficient of the plastic work converted to heat is proved as 0.9 at high strain rates. The cavitation fracture mechanism is revealed by microstructural observation over the full range explored. In basis of the experimental results and other pulished literatures, empirical Khan-Huang-Liang constitutive model was suitably modified to account for the strain-rate dependent behavior. Good agreement is achieved between the modeling prediction results and experimental data.

## 1. Introduction

α + β titanium alloys have attracted great interest in a variety of industries and fields owing to the high specific strength, reasonable ductility, and ability to withstand corrosion resistance [1,2,3]. With widespread engineering applications, the mechanical behavior of the alloys under high strain-rate loading has been essentially required not only in ballistic impact conditions, but also in normal manufacture processes [4,5,6] and the accompanied failure behavior [7,8,9]. Currently, considerable efforts have been devoted to acknowledge the rate-effect on the dynamic responses of Ti-6Al-4V alloy which has been most widely used α+β titanium alloy [10,11,12,13,14,15,16]. These studies have demonstrated that strain rate has great influence on the compressive behavior of titanium alloy. The adiabatic shear failure mechanism for titanium alloy subjected to the high strain-rate compression load has been widely accepted [12,13,14]. Few efforts have been devoted to study the tensile behavior of titanium alloy at high strain rates, despite the fact that tension-compression asymmetry has been found in the stress-strain responses and failure mechanism of α and α + β type titanium alloys [17,18,19].

The valid constitutive model has been recognized as an important factor that influences the reliability of the finite element simulation for structure design and manufacture process. In general, the flow stress is expressed as the function of strain, strain rate, and temperature, where material constants are usually determined by the interpolation of the experimental results from the constant strain-rate tests. Currently, it has been experimentally indicated that such form of constitutive model is inadequate for face-central cubic lattice metal, because of the effect of strain rate history on the strain-hardening behavior [20,21,22]. However, for α + β titanium alloys containing the hexagonal close-packed structure and body-center cubic lattice, data on the strain-rate history effect is very scarce. On the other hand, the plastic deformation process under the dynamic loading conditions is approximated as adiabatic, where the time of deformation is short and the thermal conductivity could be ignored [23]. Thus, there should be a continuous temperature rise in the specimen generated from the plastic work. It is of great significance for the accurate description of mechanical behavior to evaluate effect of the adiabatic temperature on the flow stress.

The split-hopkinson compression bar (SHPB) technique has been extensively utilized as an effective method to investigate the properties of metals at high strain rates [24]. Nemat-Nasser et al. [25] reported the novel techniques that render the classical SHPB for dynamic recovery experiments. The great progress has made it possible to conducted the direct measurement of isothermal stress-strain response of metals at high strain rates, which could effectively uncouple the adiabatic softening behavior from isothermal strain-hardening behavior. Employing this recovery technique, the variable strain rate and temperature tests also successfully came into operation, which provided effective methods to evaluate the effect of temperature and strain-rate history on the compressive behavior of metals [13,26].

TC11 titanium alloy (referenced as a kind of α + β type titanium alloy in China) has been an attracting structure material in the aviation and aerospace fields. The effect of strain rate on the tensile response of TC11 alloy has been investigated at the strain rates varying from 10^−3^ to 1150 s^−1^ and the isothermal stress-strain behaviors over a strain-rate range of 10^−3^~500 s^−1^ have been examined [27,28,29]. However, the effect of strain-rate history on tensile response has not been investigated. In addition, the modified constitutive models in the literatures [27,28] have not considered the transmission of rate sensitivity at the moderate strain-rate region. Meanwhile, it was assumed that Taylor-Quinney coefficient of the plastic work converted to heat was assumed as 0.9 and it still lack experiment evidence.

In order to improve the reliability of simulation results involving the high strain-rate deformation process, this paper mainly aims at getting a deeper insight into tensile properties and establishing the valid constitutive model of TC11 alloy over a wide range of strain rates. Employing the variable strain-rate tests, the strain rate history effect on the flow stress will be revealed. Then we will evaluate the validity of the assumption that Taylor-Quinney coefficient is taken as 0.9 based on the variable temperature tests. Finally, a phenomenologically based constitutive model is suitably modified to describe the tensile behavior observed in the current study and other published literatures of TC11 titanium alloy.

## 2. Materials and Methods

### 2.1. Material

**Figure 1:**
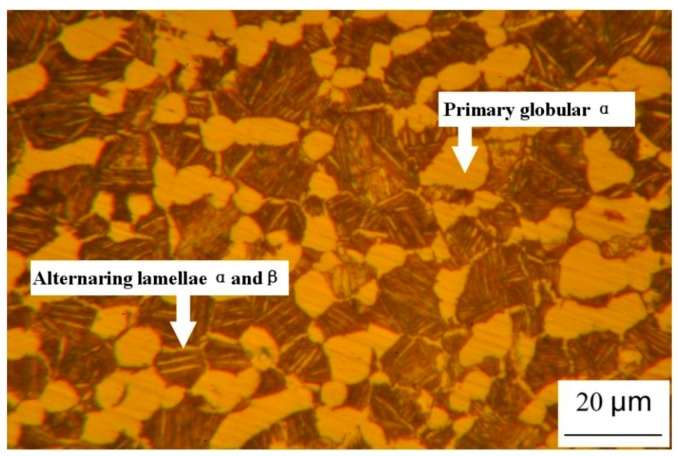
The microstructure of TC11 alloy afer the heat treatment (1228 K for 2 h+ 803 K for 6 h).

The experimental material was received as forged rods of 38 mm in diameter. The measured chemical composition (wt. %) of the alloy was listed in Table 1. The sample was fabricated to the final gage after the following sequences: (1) annealed at 1228 K for 2 h and then experienced cooling down in the air; (2) aged at 803 K for 6 h, followed by air cooling. The specimen was polished and etched with a solution of 3 mL HF + 10 mL HNO_3_ + 97 mL H_2_O. The corresponding microstructure was examined by optical microscope. The microstructure of the undeformed specimen is duplex, consisting of primary globular α grains dispersed between the lamellae Widmanstädter colony structure (seen in Figure 1).

### 2.2. Experimental Procedure

**Figure 2:**
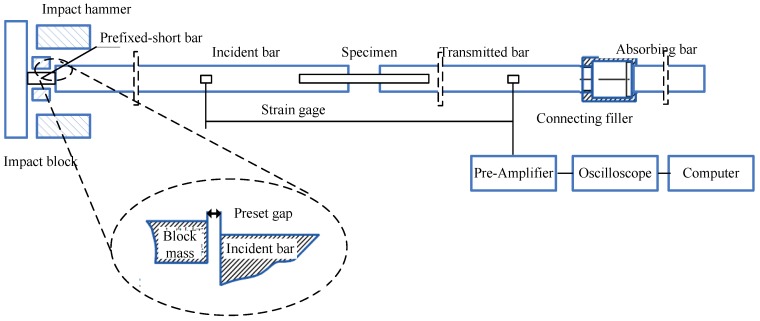
Split-hopkinson bar system for high strain-rate recovery tests.

In order to examine the history effect of strain rate on the flow stress, tension recovery experimental technique was used to carry out the variable strain-rate tests. Loading-unloading tests under quasi-static conditions (10^−3^~0.05 s^−1^) were performed using a MTS809 servo-hydraulic testing system (MTS Systems Corporation, Eden Prairie, MN, USA). Dynamic tensile tests (10^2^~1150 s^−1^) were carried out on the split-hopkinson tension bar (SHTB, Department of Modern Mechanics, University of Science and Technology of China, Hefei, China) setup. As schematically shown in Figure 2, the incident, transmitted and reflected pulses were recorded by strain gauges, which were mounted at proper places of the two bars to ensure the signal integrity. Compared with other SHTB systems, the smooth incident wave with controlled amplitude and duration was produced by the elastic-plastic deformation to fracture of the pre-fixed short metal bar [30,31].

Considering the repeated plastic deformation of the specimen by the secondary tension waves reflecting off the free ends of the bar in the original SHTB tests, the modified constructions consisting of a block mass and absorbing bar were developed to ensure the single loading-unloading tensile test. The block mass was designed at ahead of the incident bar and the absorbing bar was fixed to the end of the output bar by a connecting system, which was filled with rubber. In the well-conducted loading-unloading experiment, the precision gap must be initialized in advance between the input bar and block mass. Such gap was closed, once the entire initial tension stress pulse was transmitted into the incident bar. Consequently, the most momentum of the reflected pulse was trapped due to the interaction between the block mass and the incident bar. The width of the preset gap during the dynamic recovery experiment may be evaluated as follows:

(1) $$\Delta s=C_{0}\int_{0}^{t_{0}}\varepsilon_{i}(t)dt$$

where $\varepsilon_{i}$ is the strain history of the incident pulse and *C*_0_ represents the elastic wave speed in the bar material. 

**Figure 3:**
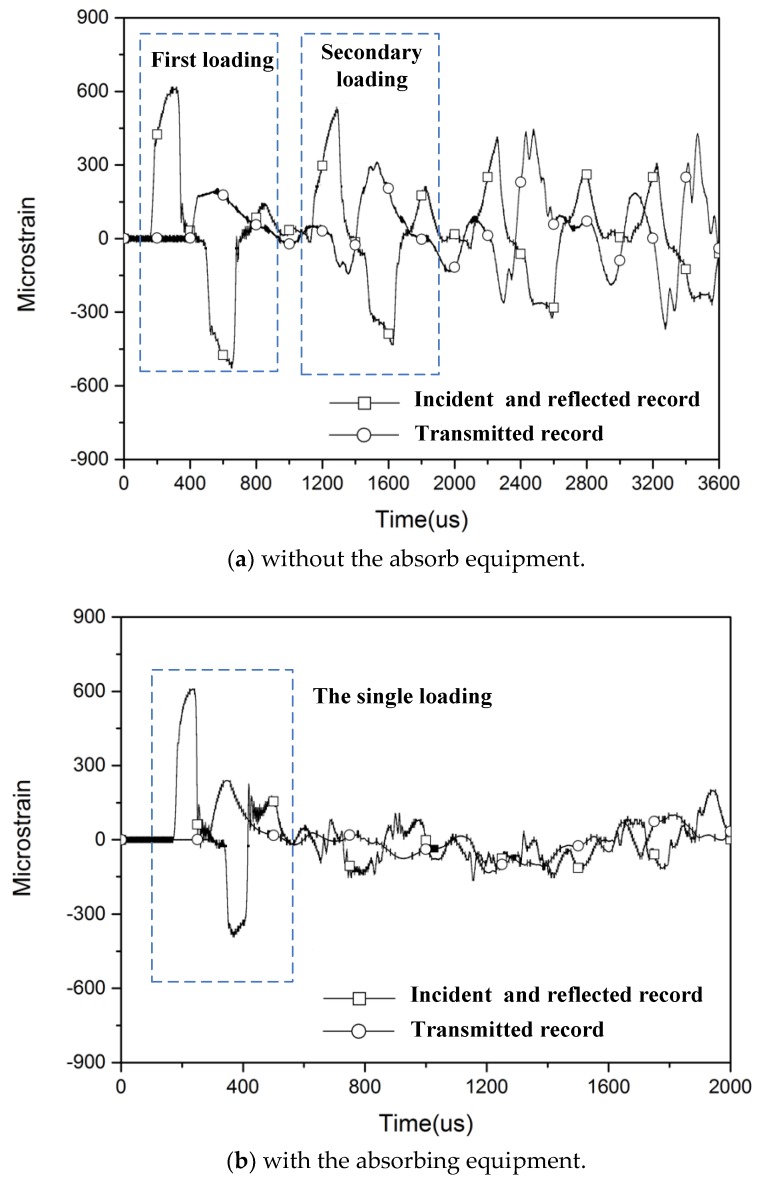
The typical signal at high strain rates.

Figure 3 shows the typical pulse signals measured by the strain gages on the incident and transmitted bar during both dynamic tension and recovery experiments. It is apparent that there exists a secondary tension loading by the reflected pulse emanating from the specimen-bar end and the specimen may experience the repeated plastic deformation in the original SHTB test. Through developing the absorbing equipment, the specimen could just withstand a single loading pulse with designed profile and recover to room temperature without any additional plastic deformation. 

The dumbbell-shape flat specimen through gluing connection with the bars was used in the dynamic tensile tests, with the gage length of 10 mm, width of 3.5 mm and fillet radius of 2 mm, respectively. The design of the adopted specimen mainly ensures the validity of one-dimensional experimental principle [32]. The specimen for low strain-rate tensile experiments was analogous except that the gage length of 30 mm is long enough to avoid the end effects.

In the present study, the deformation control routines are shown as follows:

1. A “three-step” uniaxial tensile tests were performed at low strain-rate region, where the same specimen experienced variable strain rate deformation from 10^−3^ s^−1^ to 10^−2^ s^−1^, followed by the rate of 10^−3^ s^−1^.
2. A “two-step” uniaxial tension experiments were carried out at two different strain rates. The strain rate of 10^−3^ s^−1^ was first applied until the specimen experienced a certain plastic strain. Then the deformed material was cut into the sample for dynamic test at 500 s^−1^.
3. A variable temperature tests were carried out to evaluate the adiabatic temperature rise during the high strain-rate deformation process. The specimen was first loaded to a plastic strain level at 500 s^−1^ and an initial temperature of 293 K, then recovered and deformed at higher temperature at the same stain rate. Detail discussion will be presented in the following section.

## 3. Results and Discussion

### 3.1. Effect of Strain Rate History

**Figure 4:**
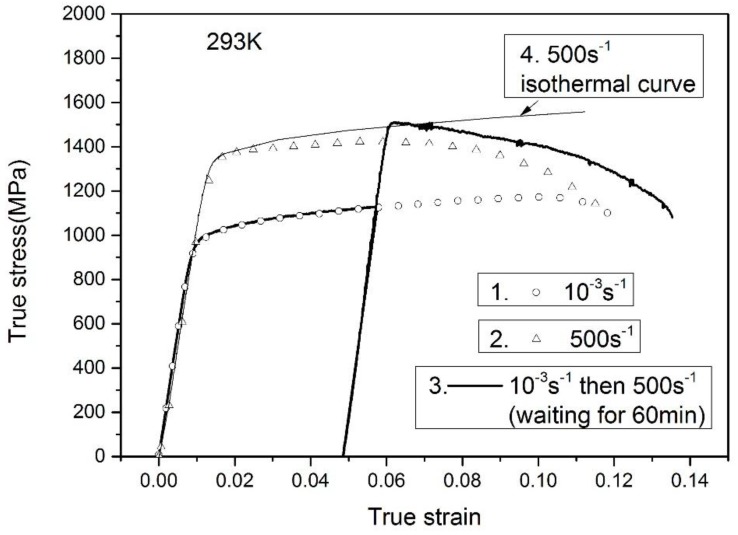
Results under variable strain-rate loading from 10^−3^ s^−1^ to 500 s^−1^.

**Figure 5:**
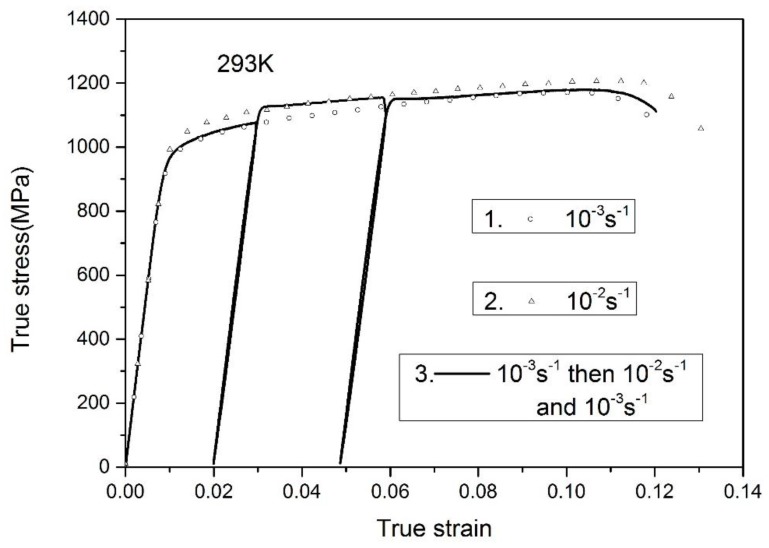
Results under variable strain rates from 10^−3^ s^−1^ to 10^−2^ s^−1^, followed by 10^−3^ s^−1^.

Figure 4 and Figure 5 depict the true stress-strain responses of TC11 titanium alloy under the constant and variable strain-rate conditions, where the true stress-strain responses are converted from the measured engineering stress and strain based on the constant volume assumption. In Figure 4, the solid lines are the variable strain-rate experimental results from 10^−3^ s^−1^ to 500 s^−1^. The symbols represent the responses under the monotonic loadings at rates of 10^−3^ s^−1^ and 500 s^−1^, respectively, along with the adiabatic and isothermal stress-strain curves at rate of 500 s^−1^ adopted from the previous literatures [27]. The isothermal stress-strain curve is achieved through connecting each initial yield stress of multi-loading-unloading curves of the same specimen. It is found that an obvious flow stress increase is generated when an increase in strain rate is applied to the deformed specimen at 10^−3^ s^−1^, where the calculated stress increase Δσ at plastic strain of 0.049 is 390 MPa. Such results indicate that the strain rate sensitivity during the strain-hardening process is consistent with that at the initial yielding period, which has been reported in the previous literature [29]. When the deformed specimen is reloaded at 500 s^-1^, the initial yield stress (adopted as the flow stress at plastic strain of 0.2%) is almost same as the flow stress of isothermal curve at the same plastic strain, rather than that of the adiabatic response. This difference is caused by the higher deformation temperature in the specimen due to the adiabatic temperature rise at monotonic loading.

In Figure 5, the solid lines are the variable strain-rate experimental results from 10^−3^ s^−1^ to 10^−2^ s^−1^, followed by the rate of 10^−3^ s^−1^. The symbols indicate the responses under the constant strain rates of 10^−3^ s^−1^ and 10^−2^ s^−1^, respectively. Similarly, the flow stress increases when the strain rate varies from 10^−3^ s^−1^ to 10^−2^ s^−1^ and decreases with the decreasing strain rate. Meanwhile, the stress-strain curves under the variable strain-rate tensile tests coincide well with the responses under the monotonous tensile tests at 10^−3^ s^−1^ and 10^−2^ s^−1^, respectively. Through conducting variable strain-rate tests, it is revealed that the strain rate history has little influence on the flow stress of Ti-6.6Al-3.3Mo-1.8Zr-0.29Si alloy. Such observation is comparable to other published experimental data of Ti-6Al-4V alloy [11]. 

### 3.2. Temperature Evolution at High Strain Rates

As stated above, it has been revealed that the adiabatic temperature rise has great influence on the plastic responses of titanium alloy. In addition, the temperature rise under high strain-rate loading condition can be determined by the following expression [33]:

(2) $$\Delta T=\frac{\eta }{\rho C_{P}}W_{p}=\frac{\eta }{\rho C_{P}}\int_{0}^{\varepsilon_{P}}\sigma d\varepsilon_{P}$$

where η is the Taylor-Quinney coefficient of the plastic work converted to heat, σ is the flow stress, and $\varepsilon_{p}$ is the plastic strain. ρ and $C_{p}$ are the mass density and the heat capacity at constant volume, respectively. For TC11 titanium alloy, the value of ρ is 4486 kg/m^3^ and $C_{p}$ is 544 J/(kg K). 

**Figure 6:**
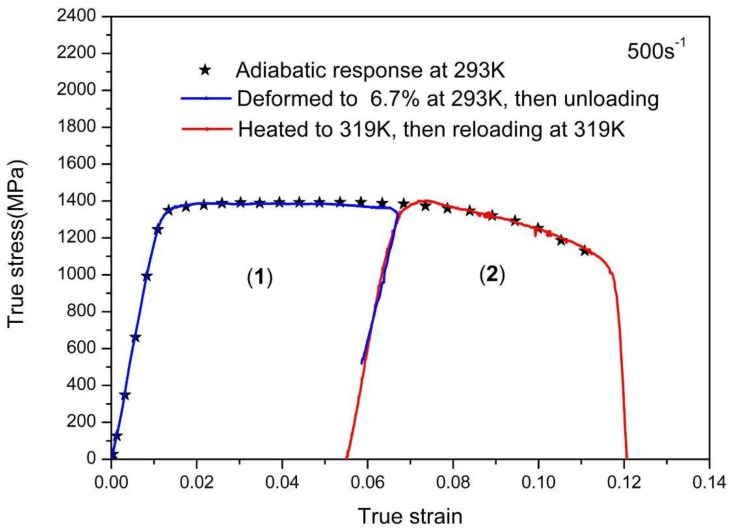
Results under the variable temperature loading.

In the previous work [27,28], the Taylor-Quinney coefficient is taken as 0.9, which indicates that 90% of the plastic work has essentially been converted into heat stored in the specimen. To verify whether η = 0.9, the temperature change experiments performed at rate of 500 s^−1^ are shown in Figure 6. The symbol line is the adiabatic curve of the TC11 alloy at the monotonous loading to fracture. Another specimen was first loaded to a plastic strain level of 6.7% at the same strain rate and an initial temperature of 293 K, then unloaded and experienced cooling down to room temperature. According to the Equation (2) and the assumption of η = 0.9, the increase of 26 K is obtained during the first deformation stage. Secondly, the specimen was heated to 319 K (namely 293 K + 26 K) using a temperature furnace and reloaded at the same strain rate of 500 s^−1^. The corresponding stress-strain curve under the reloading condition is same as the monotonic loading situation, which reveals that the assumption of η = 0.9 is good within experimental error.

### 3.3. Microstructure Analysis by OM and SEM

**Figure 7:**
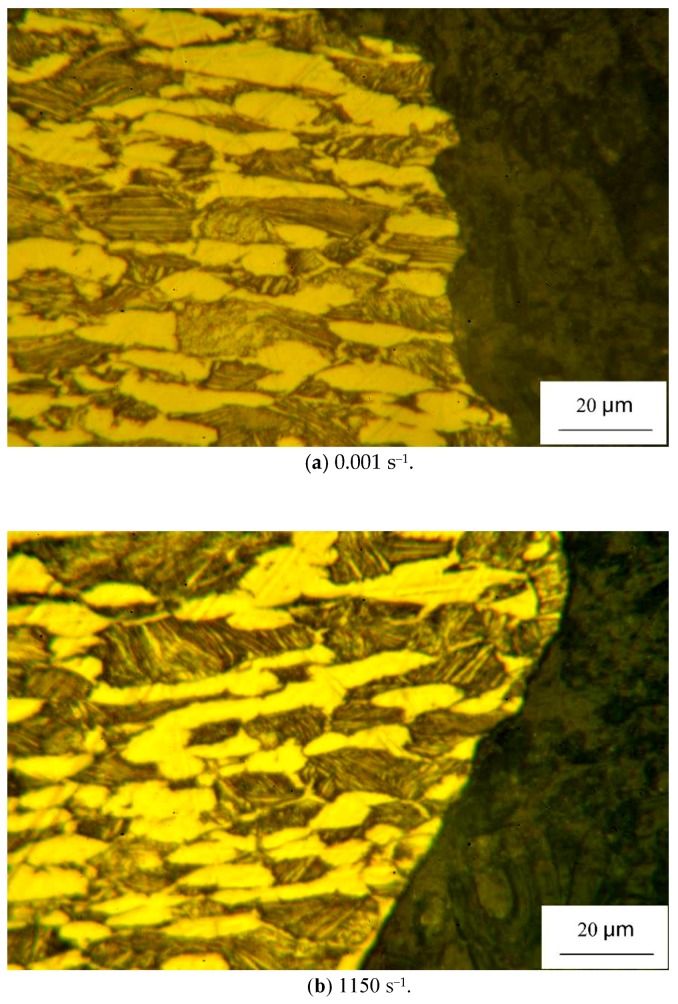
Metallographic examination of the deformed specimen at: (**a**) 10^−3^ s^−1^, (**b**) 1150 s^−1^.

**Figure 8:**
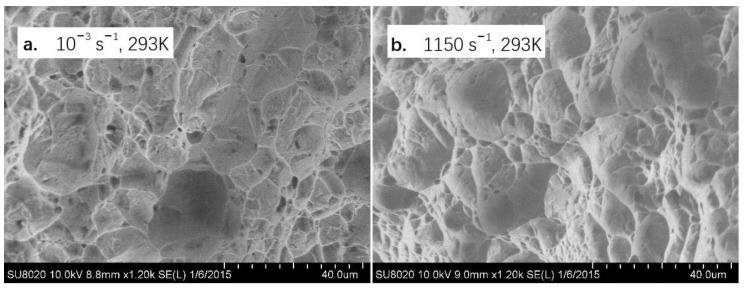
The dimple characteristics at (**a**) 10^−3^ s^−1^, (**b**) 1150 s^−1^.

The deformed specimens were carefully examined. The thickness and width of the fractured area is found to decrease, indicating the obvious necking behavior under the uniaxial tension conditions. Figure 7 depicts the microstructure besides the fracture region of the deformed sample at rate of 10^−3^ s^−1^ and 1150 s^−1^, examined by the optical microscopy (OM). The corresponding observation results of fracture surface is shown in Figure 8. The results at low and high strain rates are similar. It can be seen that the grains are apparently elongated along the tensile direction and crack randomly spreads across both lamellar Widmanstädter grains and the primary α grains. In addition, the fracture surface morphology of the cracked specimen displays typical dimple characteristics and exhibits little strain rate dependence, which reveals that the cavitation fracture is the main failure mechanism of tensile specimen over the full range explored. 

### 3.4. Constitutive Modeling of the Tension Responses

Several constitutive models have been proposed to describe the rate and thermal dependent behavior of metals. The corresponding capabilities and their inherent limitations of describing the work-hardening behavior over a wide range of strain rates have been demonstrated in the previous analysis and model correlations with experimental data [34,35]. Compared with other models, the advantage of the Khan-Huang-Liang (KHL) model is the good accommodation in the characterization of the decreasing work-hardening rate as the strain rate increases.

As already observed, the yield stress of Ti-6.6Al-3.3Mo-1.8Zr-0.29Si alloy increases with increasing strain rate, while the isothermal stress-strain curves display insensitive on the strain rate. The adiabatic temperature rise leads to the obvious softening effect on the work-hardening behavior [27,28]. The strain rate transition in the moderate strain-rate range has also been reported in detail [29]. Meanwhile, it has been revealed that the strain rate history has little influence on the flow stress. In consideration of such experimental results, the KHL constitutive model was investigated here. The flow stress in the original model is expressed as follows, consisting of the rate-thermal dependent yield stress term and the strain hardening term.

(3) $$\sigma =A{\left(\frac{\dot{\varepsilon }}{{\dot{\varepsilon }}_{*}}\right)}^{C}{\left(\frac{T_{m}-T}{T_{m}-T_{r}}\right)}^{m}+B{\left(1-\frac{\ln\dot{\varepsilon }}{\ln D_{0}^{P}}\right)}^{n_{1}}{\left(\varepsilon_{p}\right)}^{n_{0}}{\left(\frac{\dot{\varepsilon }}{{\dot{\varepsilon }}_{*}}\right)}^{C}{\left(\frac{T_{m}-T}{T_{m}-T_{r}}\right)}^{m}$$

(4) $$T=T_{0}+\Delta T$$

where ${\dot{\varepsilon }}_{*}$ is the reference strain rate, and $D_{0}^{P}$ is the upper bound strain rate. $T_{r}$ and $T_{m}$ are the reference temperature and the melt temperature of material. $T_{0}$ and ΔT are the initial environmental temperature and the temperature rise under the dynamic tension conditions. In this study, the initial environment temperature is constant, and the deformation temperature, *T*, solely relies on the temperature rise. Therefore, $T_{r}=T_{o}$ is adopted in the investigation. On the other hand, it should be noted that ${\left(\frac{\dot{\varepsilon }}{{\dot{\varepsilon }}_{*}}\right)}^{C}$ can essentially describe the constant strain rate sensitivity with the increasing logarithmic strain rates. As observed in the previous literature [], the initial yield stress has a non-linear dependence on the strain rate and appears a transition phenomenon in the moderate strain rate range. Due to such experimental observations, a modification is made to improve the accuracy of the model prediction, whose expression of the flow stress becomes:

(5) $$\sigma =[A+B{\left(1-\frac{\ln\dot{\varepsilon }}{\ln D_{0}^{P}}\right)}^{n_{1}}{\left(\varepsilon_{p}\right)}^{n_{0}}](\frac{\dot{\varepsilon }+{\dot{\varepsilon }}_{T}}{{\dot{\varepsilon }}_{*}}{)}^{C}{\left(1-\frac{\Delta T}{T_{m}-T_{r}}\right)}^{m}$$

where ${\dot{\varepsilon }}_{T}$ represents transitional strain rate.

To obtain these material parameters for the model, both adiabatic and isothermal stress-strain curves under different strain-rate loadings were first employed to achieve the initial set of values with the following steps.

1. During the initial yielding period, temperature rise and plastic strain has been identified as zero, then Equation (5) can be reduced as:

(6) $$\sigma_{s}=A{\left(\frac{\dot{\varepsilon }+{\dot{\varepsilon }}_{T}}{{\dot{\varepsilon }}_{*}}\right)}^{C}$$

The parameters in the above equation can be easily obtained by fitting the yield stresses under different strain-rate loadings.

2. As for the isothermal deformation, the flow stress is expressed as:

(7) $$\sigma_{\mathrm{isothermal}}=[A+B{\left(1-\frac{\ln\dot{\varepsilon }}{\ln D_{0}^{P}}\right)}^{n_{1}}{\left(\varepsilon_{p}\right)}^{n_{0}}]{\left(\frac{\dot{\varepsilon }+{\dot{\varepsilon }}_{T}}{{\dot{\varepsilon }}_{*}}\right)}^{C}$$

By fitting the above equation to the isothermal stress-strain experimental data in Figure 7, three material parameters, B, $n_{1}$, and $n_{2}$, can be determined.

3. By comparing the isothermal and adiabatic flow stress at different strains, the remaining constant, *m*, can be approximately evaluated. The corresponding derivation is:

(8) $$m=\ln(\frac{\sigma_{\mathrm{adiabatic}}}{\sigma_{\mathrm{isothermal}}})/\ln(1-\frac{\Delta T}{T_{m}-T_{r}})$$

**Figure 9:**
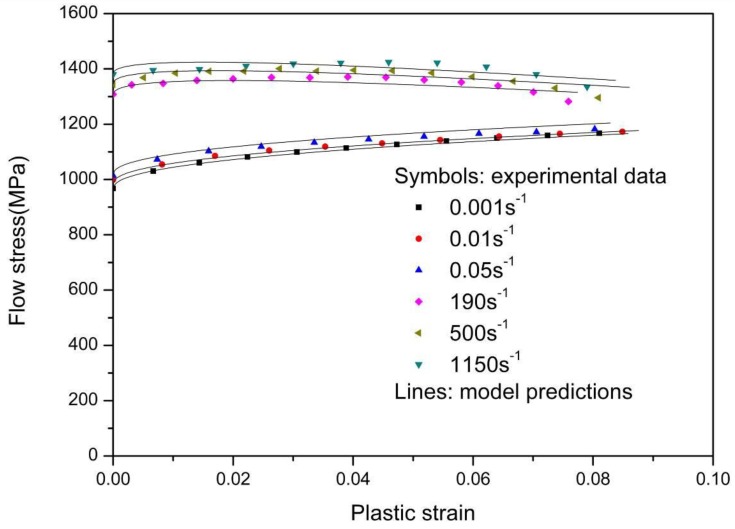
Comparison of model prediction with experimental results under different strain rates.

For the TC11 alloy, the melting temperature is 1933 K. The reference strain rate and upper bound strain rate are adopted as 10^−3^ s^−1^ and 10^6^ s^−1^, respectively. The reference temperature is the initial environmental temperature, whose value is 293 K. Table 2 shows the simulated values of the material parameters. The comparison between the modified model predictions and experimental flow stress-plastic strain responses is displayed in Figure 9. It can be seen in Figure 9 that the results of model prediction coincide reasonably well with the experimental data. These results indicate that the modified KHL model is suitable to describe the tensile behavior of TC11 titanium alloy over a wide range of strain rates. 

## 4. Conclusions

In this study, the stress-strain responses of TC11 titanium alloy under a variety of strain rates from 10^−3^ s^−1^ to 500 s^−1^ were investigated. The modified Khan-Huang-Liang constitutive model was suitably proposed. The conclusions were drawn as follows:

(1) An increase of flow stress is observed under the variable strain-rate loading from 10^−3^ s^−1^ to 500 s^−1^, indicating the obvious strain-rate strengthening behavior during the strain-hardening process;
(2) The stress-strain curves under the variable strain-rate tensile tests coincide well with the responses under the monotonous tensile tests at the same strain-rate, plastic strain and temperature. It is revealed that the strain rate history has little influence on the flow stress of Ti-6.6Al-3.3Mo-1.8Zr-0.29Si alloy;
(3) By conducting the variable temperature tests, the Taylor-Quinney coefficient is proved as 0.9 at high strain rate;
(4) The Khan-Huang-Liang constitutive model is modified to improve the ability to describe the strain rate transition of flow stress and adiabatic softening effect. Based on the experimental results and other published data obtained from the recovery tests, an effective way to determine the material constants is presented. Good agreement is achieved between the model predictions and the experimental data;
(5) The cavitation fracture mechanism is revealed by microstructural observation over the full range explored. Further, a crystal plasticity model incorporating the microstructure evolution will be studied in the future.

## Figures and Tables

**Table 1:** Chemical composition (wt. %) of TC11 alloy.

Al	Mo	Zr	Si	Fe	C	N	H	O	Ti
6.6	3.3	1.8	0.29	0.07	0.01	0.01	0.004	0.13	balance

**Table 2:** Values of the modified KHL model constants for TC11.

*A*(MPa)	*B*(MPa)	*n*_1_	*n*_0_	*C*	*m*	${\dot{\varepsilon }}_{T}(s-1)$
895	438	0.52	0.43	0.031	4.65	0.01
